# Infodemiology and Infoveillance of the Four Most Widespread Arbovirus Diseases in Italy

**DOI:** 10.3390/epidemiologia5030024

**Published:** 2024-07-05

**Authors:** Omar Enzo Santangelo, Sandro Provenzano, Carlotta Vella, Alberto Firenze, Lorenzo Stacchini, Fabrizio Cedrone, Vincenza Gianfredi

**Affiliations:** 1 Regional Health Care and Social Agency of Lodi, ASST Lodi, 26900 Lodi, Italy; omarenzosantangelo@hotmail.it (O.E.S.); carlotta.vella@asst-lodi.it (C.V.); 2 Faculty of Medicine, University of Milan, 20133 Milan, Italy; 3 Local Health Unit of Trapani, ASP Trapani, 91100 Trapani, Italy; 4 Department of Health Promotion, Mother and Child Care, Internal Medicine and Medical Specialties (PROMISE), University of Palermo, 90133 Palermo, Italy; alberto.firenze@unipa.it; 5 Department of Health Science, University of Florence, 50134 Florence, Italy; lorenzo.stacchini@unifi.it; 6 Local Health Unit of Pescara, Hospital Management, 65122 Pescara, Italy; cedronefab@gmail.com; 7 Department of Biomedical Sciences for Health, University of Milan, Via Pascal, 36, 20133 Milan, Italy; vincenza.gianfredi@unimi.it

**Keywords:** Toscana phlebovirus, West Nile virus, tick-borne encephalitis, dengue, infodemiology, infoveillance

## Abstract

The purpose of this observational study was to evaluate the potential epidemiological trend of arboviral diseases most reported in Italy by the dedicated national surveillance system (ISS data) compared to searches on the internet, assessing whether a correlation/association between users’ searches in Google and Wikipedia and real cases exists. The study considers a time interval from June 2012 to December 2023. We used the following Italian search terms: “Virus Toscana”, “Virus del Nilo occidentale” (West Nile Virus in English), “Encefalite trasmessa da zecche” (Tick Borne encephalitis in English), and “Dengue”. We overlapped Google Trends and Wikipedia data to perform a linear regression and correlation analysis. Statistical analyses were performed using Pearson’s correlation coefficient (r) or Spearman’s rank correlation coefficient (rho) as appropriate. All the correlations between the ISS data and Wikipedia or GT exhibited statistical significance. The correlations were strong for Dengue GT and ISS (rho = 0.71) and TBE GT and ISS (rho = 0.71), while the remaining correlations had values of r and rho between 0.32 and 0.67, showing a moderate temporal correlation. The observed correlations and regression models provide a foundation for future research, encouraging a more nuanced exploration of the dynamics between digital information-seeking behavior and disease prevalence.

## 1. Introduction

Vector-borne diseases constitute an important public health issue as the World Health Organization (WHO) estimates that every year, they cause over 1 billion human cases and 1 million deaths, representing approximately 17% of the total cases of communicable diseases [1]. The distribution of vector-borne diseases is determined by a complex set of demographic data, environmental, and social factors, and many of them are preventable through protective measures and community mobilization [1]. Among the vector-borne diseases, an important group is made up of arboviruses, which are a complex group of RNA viruses transmitted by arthropods. Arthropod-borne viral (arboviral) diseases are zoonoses caused by arthropod-borne viruses, and they affect both humans and animals. Viruses are spread through the bite of infected vectors, such as mosquitoes, ticks, and sand flies [2]. Arboviruses are a diverse group of vector-borne viruses, many of which are the cause of significant human morbidity and mortality. There are >500 recognized arboviruses worldwide, 150 of which are known to cause disease in humans [3]. Most zoonotic arboviruses belong to two major families, namely Flaviviridae (Flavivirus) and Togaviridae (Alphavirus), as well as the order Bunyavirales (Bunyavirus and Phlebovirus). Also, more families have demonstrated pathogenicity in humans, including the Reoviridae, Rhabdoviridae, and Orthomyxoviridae families.

Symptoms of arboviral infections can range from very mild to very severe and usually appear 3 to 14 days after a bite from an infected mosquito or tick but can vary depending on the specific infection. According to the Italian National Institute of Health (Istituto Superiore di Sanità, ISS), arboviruses are responsible for indigenous or imported infections, e.g., in the case of international travelers who visit risky places. Most infected people are asymptomatic or have mild symptoms, such as mild fever, headache, muscle or joint pain, and/or skin rash, which resolve without serious health problems. Severe infections are characterized by rapid onset, headache, hemorrhagic fever, polyarthralgia, encephalitis, confusion, tremors, convulsions, paralysis, coma, or death. Arboviral diseases should therefore be considered in differential diagnosis when patients have a history of travel or if certain arboviruses are known to be spreading across the country [2], according to the National Plan for Prevention, Surveillance, and Response to Arboviruses. In Italy, there are native arboviruses (including the West Nile Virus, Toscana virus infection, and Tick-borne encephalitis) and mainly imported arboviruses, such as infections caused by viruses Chikungunya, Dengue and Zika [4]. Although the lifetime burden of arboviral diseases on many countries is still poorly studied, often both at a national and international level, we have witnessed several epidemic events, sometimes of significant dimensions [5]. As mentioned, clinical manifestations can range from mild to severe, and the variability in terms of pathogens makes the appropriate diagnosis difficult. Therefore, understanding the epidemiology, clinical presentation, pathogenesis, and management of arboviral diseases is essential for prevention, diagnosis, and treatment. In this context, the National Surveillance System (NSS) has acquired particular attention. The NSS is a nationwide collaboration that enables all levels of public health (local, state, territorial) to share health information to monitor, control, and prevent the occurrence and spread of state-reportable and nationally notifiable infections [6]. In Italy, regarding the arboviral diseases, surveillance plans are in place specifically for the following viruses: Chikungunya, Dengue, Zika, West Nile Virus (WNV), Usutu, Tick-borne encephalitis (TBE), and neuroinvasive infections caused by the Toscana virus (TOSV). The integrated surveillance of arboviral diseases in Italy is coordinated by the ISS in collaboration with the Ministry of Health, which regularly publishes surveillance and response plans to ensure the early detection of potential cases and minimize any spread of disease [2,4]. Last year, between 1 January and 31 December 2023, as per the monthly update, the national surveillance system recorded the following [7]:

- Up to 50 confirmed cases of neuroinvasive infection—TBE (47 local cases and 3 linked to a trip abroad, with a median age of 58.5 years; 70% males; no deaths);
- A total of 362 confirmed cases of Dengue (82 local cases and 280 linked to trips abroad, with a median age of 37 years; 52% males; one death);
- Approximately 127 confirmed cases of infection with Toscana virus (125 local cases, with a median age of 52 years; 65% males; no deaths);
- Nine confirmed cases of Zika Virus (all linked to trips abroad, with a median age of 30 years; 44% males; no deaths);
- Seven confirmed cases of Chikungunya (all linked to trips abroad, with a median age of 42 years; 71% males; no deaths);
- And over 330 cases of West Nile fever.

Since the beginning of the century, new opportunities, enabled by the underlying availability and scale of internet-based sources (IBSs), have paved the way for novel approaches to public health knowledge. Internet-based sources have proven to serve as a crucial resource for analyzing trends of various health-related topics, serving as a method to assess the public interest in them. The use of these sources for public health purposes is also known as “infodemiology” (from union of information and epidemiology) or “infoveillance” (from fusion of information and surveillance) (Eysenbach 2009), and this can be anticipated to have a positive impact on analysis within the healthcare field. Further fields of study could be focused on to study the existence of the correlation between real infectious disease data and online search traffic data.

Considering all the above, we performed this cross-sectional analysis aimed at assessing the potential epidemiological trend of the arboviral diseases most reported in Italy by the dedicated national surveillance system (i.e., Dengue, Toscana virus, Tick-borne encephalitis and West Nile Virus) compared to searches on the internet, assessing whether a correlation/association between the users’ searches in Google and Wikipedia and real cases exists.

## 2. Materials and Methods

This cross-sectional study considers the period of time from June 2012 to December 2023, with variations in the time interval based on the virus under consideration and the available data. The real cases of disease were extracted from the Italian National Institute of Health, Istituto Superiore di Sanità, in Italian (ISS) [8]. The ISS publishes monthly bulletins regarding the reported cases of arbovirus in Italy [8,9]. The study considered data that included all confirmed cases, both indigenous and imported. Considering the data reported since the beginning of their publication, the following time intervals were extracted for the four arboviruses considered: Dengue from January 2015 to December 2023, the Toscana virus (TOSV) from January 2016 to December 2023, Tick-borne encephalitis (TBE) from January 2016 to December 2023, and the West Nile Virus (WNV) from June 2012 to December 2023. Similarly, for the same time intervals, the internet search data by users were extracted for the same search topics using Google Trends (GT) in order to be able to match the ISS data with those of GT, in particular the relative search volume (RSV) was extracted [10]. GT provides an index called RSV, where the values can range between 100 and 0. This value represents a percentage calculated based on the proportion of a specific term for a given location and time period. Regarding Wikipedia, through a specific tool, the authors were able to identify the number of views of a specific page by users in the desired time interval [11]. From Wikipedia, we extracted the number of times the following Italian pages were viewed: “Virus Toscana”, “Virus del Nilo occidentale” (West Nile Virus in English), “Encefalite trasmessa da zecche” (Tick Borne encephalitis in English) and “Dengue”, and then we aggregated the data monthly to make them comparable; the extraction period goes from the beginning (data available from July 2015) to December 2023. The above-mentioned keywords were not selected; instead, we directly assessed all the unique Italian Wikipedia pages related to the diseases under consideration. Statistical analyses were performed using Pearson’s correlation coefficient (r) or Spearman’s rank correlation coefficient (rho) as appropriate. The correlation was considered strong if the coefficient was >0.7, moderate if between 0.3 and 0.7 and weak if <0.3 [12]. Linear regression models were performed, considering the number of views of Wikipedia pages and Google RSV as dependent variables, while the independent variables were the notified cases of illness. Results were expressed as a coefficient with 95% confidence intervals (95% CI). Potential autocorrelation was ascertained by calculating the Durbin–Watson (DW) statistic, a test used to determine the existence of autocorrelation in the residuals (prediction errors) from a regression analysis. The DW or *d* test statistic always ranges from 0 to 4. If *d* is significantly less than 2, it indicates positive serial correlation, whereas values greater than 2 imply the absence of autocorrelation. The representative linear model was also determined by calculating the R^2^ of the model. The analyses were conducted with a statistical significance level of 0.05 using the STATA statistical software, version 14, and Microsoft Excel^®,^ version 2016. The data download and analyses were conducted on the 20 February 2024.

## 3. Results

**Figure 1:**
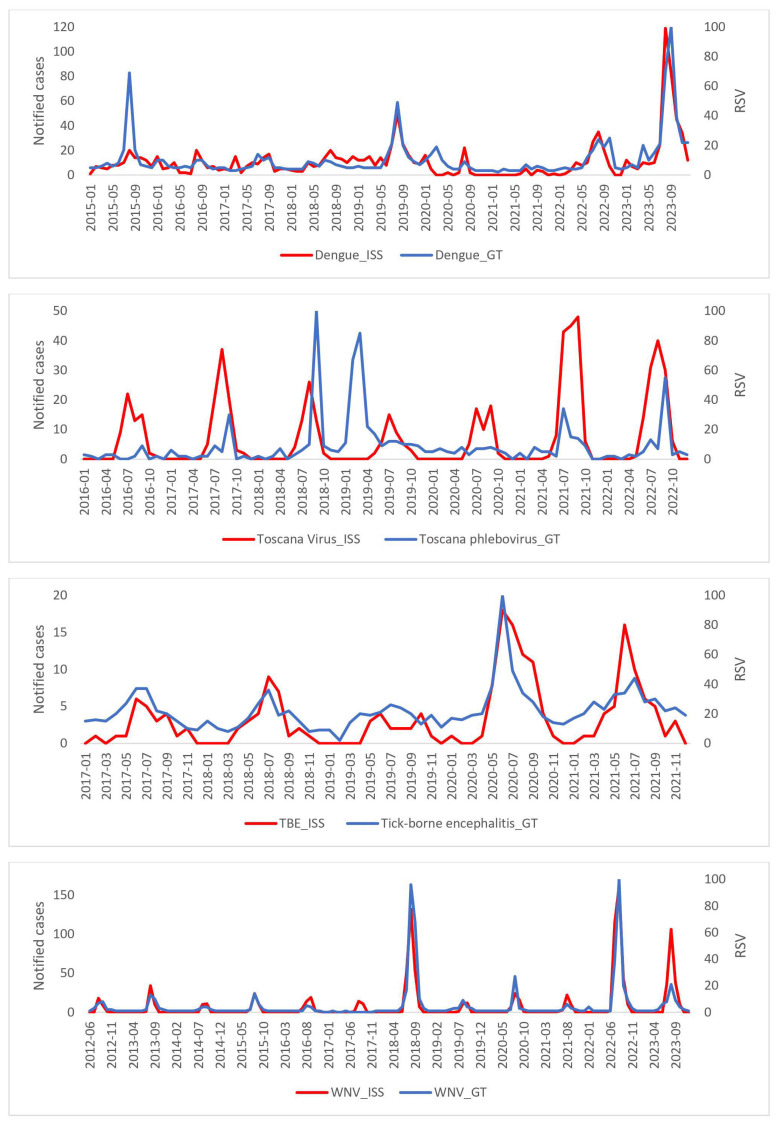
Search trend of Google RSV and ISS notified cases.

**Figure 2:**
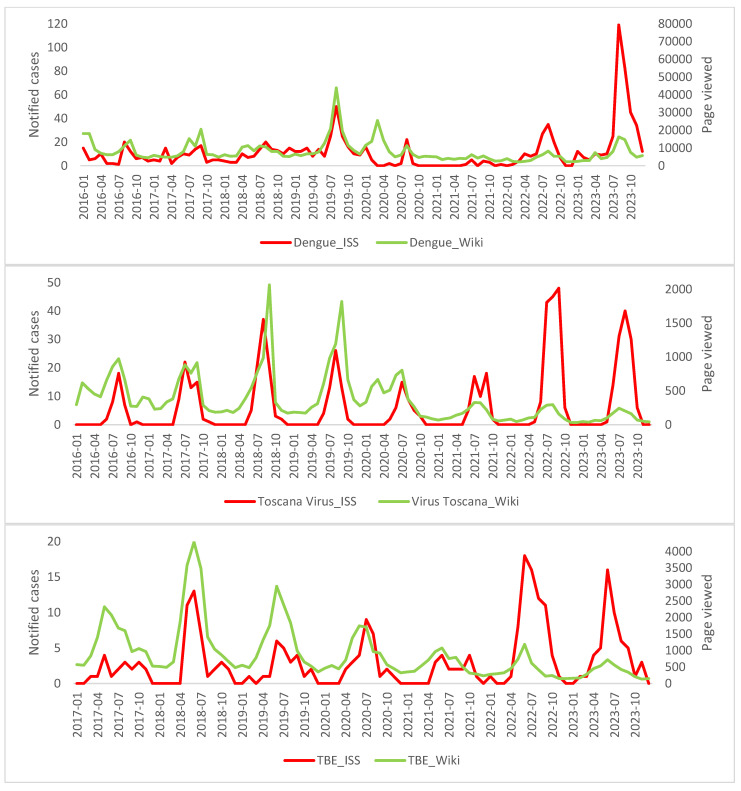

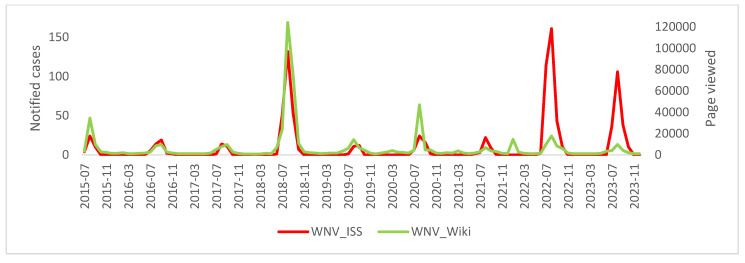
Search trend of Wikipedia page viewed and ISS notified cases.

**Figure 3:**
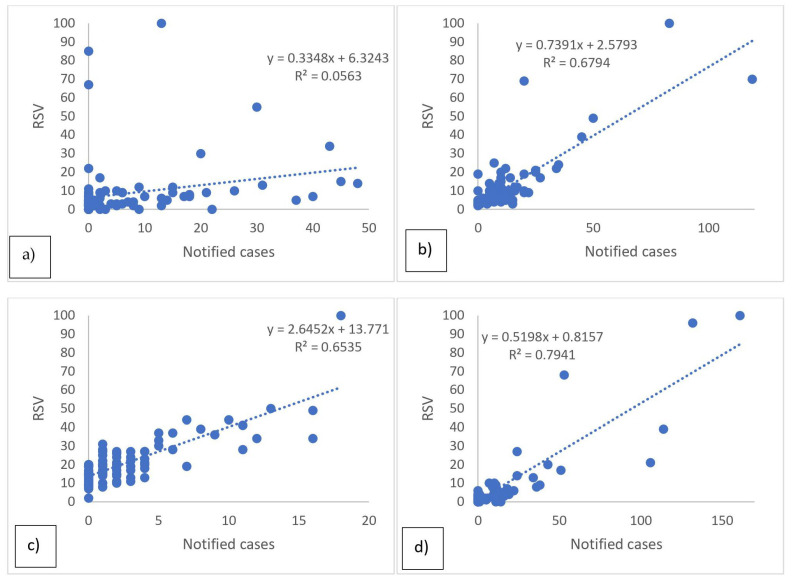
Linear regression models between notified cases of ISS and Google’s RSV. Dengue (**a**), TOSV (**b**), TBE (**c**), WNV (**d**).

**Figure 4:**
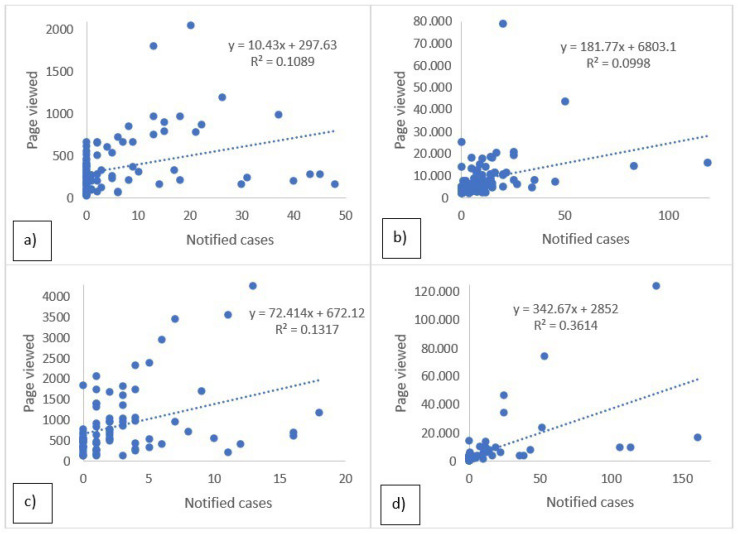
Linear regression models between notified cases of ISS and Wikipedia searches. Dengue (**a**), TOSV (**b**), TBE (**c**), WNV (**d**).

As for the raw search data for Wikipedia, GT and ISS are shown in Figure 1 and Figure 2, where the peaks between the searches and the real cases of disease are generally superimposable. Table 1 shows the results of the temporal correlations between the cases notified by the ISS and the related pages viewed on Wikipedia or the Google RSV. All correlations exhibit statistical significance temporal correlations between ISS data and Wikipedia or GT. The correlations are strong for Dengue GT and ISS (rho = 0.71), and TBE GT and ISS (rho = 0.71), suggesting a robust link between the occurrences of these diseases and online information-seeking patterns. For the six remaining correlations, the values of r and rho are between 0.32 and 0.67 thus showing a moderate temporal correlation. Table 2 shows the results of the calculated linear regression models which also indicate that, in this case, all the regressions are statistically significant, specifically in Figure 3 and Figure 4. In the linear regression models between the ISS cases and the pages viewed in Wikipedia and the ISS and Google RSV cases, the GT–ISS models generally have a higher R2 than the Wikipedia–ISS models, except in the case of Dengue which is probably due to some outliers (which may have been caused by greater media attention in certain periods). 

## 4. Discussion

### 4.1. Data Interpretation

The current study provides valuable insights into the temporal correlations and linear regression models between the cases notified by the ISS, Wikipedia page views, and GT. These findings offer a deeper understanding of the relationships between online information-seeking behavior and the reported cases of diseases, shedding light on the potential utility of digital data in disease surveillance. In particular, the results reveal significant temporal correlations between the ISS data and both the Wikipedia page views and GT. Specifically, strong correlations were found for Dengue GT and ISS, and TBE GT and ISS. Moderate temporal correlations were observed for the remaining data, with correlation coefficients which indicate a robust link between these diseases and online information-seeking behavior. These findings imply that there is a discernible association between the frequency of disease cases reported by the ISS and the corresponding online search behavior. Notably, the strong correlations for Dengue and TBE suggest that these diseases might receive more attention in the media and public discourse, leading to increased online search activity. This heightened attention could be due to several factors. For instance, Dengue fever might be given more media coverage in Italy, driving public interest and search activity. Official statements by the health authorities about the risk and incidence of Dengue and TBE could also amplify search behavior. Conversely, diseases like the Toscana virus may not receive as much media attention or public health communication, resulting in lower search activity. Additionally, the level of public awareness and education regarding the risks of specific diseases could influence search behavior. Insufficient educational efforts and low diagnostic levels (underdiagnosis) may contribute to an underestimated incidence and lower search volumes for diseases like the Toscana virus.

It is noteworthy that the moderate correlations still demonstrate a significant connection, indicating that online search patterns may provide useful supplementary information for disease surveillance. However, the nature of this correlation warrants further investigation to uncover the underlying factors influencing the observed trends. In fact, various underlying factors might influence the correlation between cases of infectious diseases reported to traditional surveillance systems and the internet search volumes. Among them, increased media coverage can drive public interest and therefore online search activity. Consequently, the augmented search activity may reflect higher awareness during disease outbreaks. Similarly, official announcements by health authorities, government agencies, or health awareness campaigns and public education initiatives related to specific infectious diseases can influence search behavior. On the other hand, even rumors or misinformation about health conditions (or infectious diseases) can lead to increased online searches. At the same time, social and cultural factors, such as celebrities’ announcement of a disease or celebrities’ appeal on health-related issues may impact the way people search for information about health. Nevertheless, the availability and accessibility of technology, including internet access and smartphones, can influence the ease with which individuals seek information online. Understanding these factors is crucial for interpreting the observed correlations accurately.

Furthermore, when considering the linear regression models, the results reinforce the observed temporal correlations, revealing statistically significant relationships between ISS cases and online search data. Notably, the linear regression models between ISS cases and GT generally exhibit higher R2 values compared to the models involving Wikipedia page views. This suggests that GT might be a more reliable predictor of disease incidence, except for Dengue, where potential outliers might be influencing the accuracy of the model. The presence of outliers in the Dengue model underscores the importance of cautious interpretation, as sudden spikes in media attention can introduce distortions in the data. This anomaly emphasizes the need for robust data cleaning and consideration of external factors influencing search behavior during analysis.

Moreover, when plotted, the raw search data for Wikipedia, Google RSV, and ISS further emphasize the synchronicity between online search peaks and actual disease cases. The overlapping peaks indicate that increases in online information-seeking align with peaks in disease incidence, supporting the notion that digital data sources can act as early indicators of disease outbreaks.

In 2023, for Dengue, the GT research indicates an increase in searches. According to the authors, this increase may be due to multiple factors. Compared to previous years, there have been autochthonous cases (20% of the total), whereas Dengue has generally been an imported disease. This has heightened media attention on the disease and may have influenced the number of searches. Additionally, the approval of a new vaccine against Dengue could have intrigued internet users, prompting them to research the disease [13]. Finally, the number of Dengue cases in 2023 was significantly higher than in previous years, and generally, as the number of cases increased, the number of searches also increased.

Another disease that recorded a similar number of cases to Dengue is WNV but considering that 99% of the cases during the study period were indigenous, there was not the same level of media attention as for Dengue.

In summary, the data presented in this study confirm what we previously explored, when the data from the national surveillance system for the period 2015–2019, concerning cases of Dengue and the West Nile Virus, were associated with only the search volumes on Wikipedia [14]. In this study, in addition to extending the temporal reference range until 2023, we also considered the search volumes on Google and two other arboviruses (Toscana virus and TBE).

### 4.2. Implication for Policies and Practices

Results of the current research provide important implications for policies and practices. In fact, although digital epidemiology is not yet prepared to substitute traditional surveillance systems [15], it can represent a valuable supportive tool. Integrating traditional surveillance systems with the analysis of user search trends on platforms like Google and Wikipedia could assist political decision-makers in implementing preventive measures and information programs for the population [15,16]. This field of study is still in its infancy and relatively unexplored, but it is likely to experience rapid growth, particularly due to the ease of accessing data, its increasing availability, and the computational power of computers [17].

It is important to note that surveillance systems are not in real-time, whereas internet searches occur in real-time. A surge in searches during a specific period and in a particular geographical location could serve as an early “alert” thus warranting attention [18,19]. This approach could potentially be used in the near future as a supplementary tool for predicting epidemics, for instance [15,17,20]. This approach could aid in forecasting disease trends and help public health agencies allocate resources more effectively [21]. Moreover, developing real-time monitoring systems based on digital data can enhance the agility of public health interventions. Additionally, further studies are advisable to comprehend how to effectively integrate, analyze, and utilize this data in an intelligent, prompt, and beneficial manner, and how to communicate them adequately [22,23]. Lastly, given the global nature of infectious diseases, international collaboration and standardization of methodologies are crucial [24]. Future research can contribute to the development of standardized approaches for digital epidemiology, facilitating cross-country comparisons and collaborative efforts [25].

### 4.3. Future Perspectives in Research

The results shown in the current manuscript offer important perspectives for future research. First, the strong temporal correlations observed suggest that online search data could serve as an early warning system for infectious disease outbreaks and therefore future research can focus on refining models to improve the accuracy of early detection, enabling more timely public health interventions [26,27]. Particularly, further research and analysis are needed to tease apart the individual contributions of these factors and to develop robust models for effectively utilizing online search data in disease surveillance [28,29,30]. Additionally, ongoing monitoring and adaptation of the methodologies will be essential as the landscape of online information and public health evolves [31,32]. Second, future research should enhance the methodologies aimed at integrating traditional surveillance systems with digital data sources [20,33]. Indeed, combining multiple datasets contribute to the creation of comprehensive and complementary disease surveillance systems that offer a more holistic view of public health trends [34]. Third, future research can explore the impact of public health interventions, media campaigns, or awareness initiatives on online search patterns [35,36]. Such data could inform more targeted and effective public health strategies. Fourth, more validation studies assessing the robustness of the observed correlations across different infectious diseases and geographic regions are essential in order to establish the generalizability and reliability of this approach [37,38,39]. Lastly, being capable of building predictive models that leverage the observed correlations can be a future area of research as well.

### 4.4. Limitations and Strengths

This study is not without limitations. Specifically, we did not take into account potential media events, search engine algorithms, and demographic disparities in internet access that could have influenced the internet research volume. Moreover, due to the cross-sectional nature of the study, causality cannot be assessed. Further, we only utilized data from both GT and Wikipedia; however, it is crucial to acknowledge that these sources do not encompass all the available options for users looking for information on the internet. This study did not consider other search engines like Yahoo! or Bing, nor did it include data from social media platforms. However, existing research indicates that over 80% of internet users worldwide utilize Google [40]. Even though GT lacks information about user characteristics, making it challenging to profile individuals searching for specific topics, these innovative data sources still present scientists and policymakers with several opportunities. Moreover, the possibility of selection bias is real, considering that Google users do not necessarily represent the entire population. Similarly, there is the possibility of self-selection bias, where searches on other search engines may be excluded. This potential bias, which could distort the results, was addressed by setting up a comparison between Google Trends, Wikipedia, and data from the ISS, implementing a real external validation process. Regarding possible measurement biases due to the ambiguity of search terms, the specificity of the words searched and described in the Materials and Methods seems to significantly reduce this risk. Another important risk of bias in this type of study is the temporal type. However, in this specific case, a sufficiently long observation period was used to capture long-term trends, thereby reducing the impact of seasonal or temporary events.

Analyzing new data streams has paved the way for research in multiple fields, including acute diseases, emerging or re-emerging infectious diseases, and chronic conditions such as cancer [41].

Another limitation of the study is that it does not distinguish searches based on autochthonous and imported cases. Currently, there are technical limitations that prevent the differentiation of the types of users. However, for Dengue, it is worth pointing out that there have been indigenous cases which could have positively impacted the number of searches, but this was limited to the year 2023, within our observation period (20% of cases are autochthonous and confined to two regions, Lombardy and Lazio). The study considered the period from 2015 to 2023. Furthermore, as of 19 June 2024, no autochthonous cases have been recorded for 2024, suggesting that it could have been a rare occurrence in 2023. Therefore, it is plausible to think that the influence of autochthonous Dengue cases on searches during the study period is minimal.

To provide context and interpret the findings, considering the mentioned limitations, this innovative approach should be supplemented and supported by traditional analytical methods, such as surveys or evaluations of medical data. Combining these approaches ensures a more comprehensive and robust understanding of the dynamics surrounding online health-related information seeking.

Nevertheless, our study has some strengths as well. This can be considered as a pilot study that offers a new point of view regarding digital surveillance systems and sheds light on the impact of analyzing this data in the public health area.

## 5. Conclusions

In conclusion, the presented findings highlight the potential of online search data, particularly Google RSV, as a valuable tool in disease surveillance. The observed correlations and regression models provide a foundation for future research, encouraging a more nuanced exploration of the dynamics between digital information-seeking behavior and disease prevalence. Further investigations should aim to elucidate the causative factors behind these correlations and address potential limitations, such as outliers and external influences, to enhance the reliability and applicability of digital surveillance tools.

## Figures and Tables

**Table 1:** Spearman’s rank correlation coefficient (rho) or Pearson’s correlation coefficient (r) between cases reported by ISS and the visualization of the Wikipedia page or RSV Google Trends in Italy.

	Dengue GT		Dengue Wikipedia	
Dengue ISS	rho	0.71	r	0.32
	*p*-value	<0.001	*p*-value	0.001
	observations	108	observations	102
	**TOSV GT**		**TOSV Wikipedia**	
TOSV ISS	rho	0.40	r	0.33
	*p*-value	<0.001	*p*-value	0.001
	observations	96	observations	96
	**TBE GT**		**TBE Wikipedia**	
TBE ISS	rho	0.71	r	0.36
	*p*-value	<0.001	*p*-value	<0.001
	observations	84	observations	84
	**WNV GT**		**WNV Wikipedia**	
WNV ISS	rho	0.67	r	0.60
	*p*-value	<0.001	*p*-value	<0.001
	observations	139	observations	102

Italian National Institute of Health—Istituto Superiore di Sanità: ISS, West Nile Virus: WNV, Tick Borne Encephalitis: TBE, Toscana virus: TOSV, Google Trends: GT.

**Table 2:** Linear regression models.

	Dependent variable: Dengue GT				Dependent variable: Dengue Wikipedia			
Independent variable	Coefficient	95% CI	*p*-value	Durbin Watson	Coefficient	95% CI	*p*-value	Durbin Watson
Dengue ISS	0.74	0.64–0.84	<0.001	1.80	181.77	73.45–290.10	0.001	1.91
	**Dependent variable: TOSV GT**				**Dependent variable: TOSV Wikipedia**			
Independent variable	Coefficient	95% CI	*p*-value	Durbin Watson	Coefficient	95% CI	*p*-value	Durbin Watson
TOSV ISS	0.33	0.05–0.62	0.020	1.43	10.43	4.32–16.54	0.001	0.69
	**Dependent variable: TBE GT**				**Dependent variable: TBE Wikipedia**			
Independent variable	Coefficient	95% CI	*p*-value	Durbin Watson	Coefficient	95% CI	*p*-value	Durbin Watson
TBE ISS	2.65	2.22–3.07	<0.001	1.63	72.41	31.56–113.26	<0.001	0.27
	**Dependent variable: WNV GT**				**Dependent variable: WNV Wikipedia**			
Independent variable	Coefficient	95% CI	*p*-value	Durbin Watson	Coefficient	95% CI	*p*-value	Durbin Watson
WNV ISS	0.52	0.48–0.56	<0.001	1.47	342.67	252.30–433.04	<0.001	1.04

Italian National Institute of Health—Istituto Superiore di Sanità: ISS, West Nile Virus: WNV, Tick Borne Encephalitis: TBE, Toscana virus: TOSV, Google Trends: GT.

## Data Availability

All data are published.
